# Antibody Responses to EBV and Toxoplasma and Their Genetic Links to Guillain–Barré Syndrome: A Mendelian Randomization Study

**DOI:** 10.1002/brb3.70298

**Published:** 2025-02-09

**Authors:** Xiangjia Qi, Liqian Gao, Lifeng Qi

**Affiliations:** ^1^ Department of Neurology Liaocheng People's Hospital Liaocheng China

**Keywords:** antibody immune responses, Guillain–Barré syndrome, two‐sample Mendelian randomization

## Abstract

**Background:**

This study aims to investigate the potential causal relationship between antibody‐mediated immune responses to infectious agents and Guillain–Barré syndrome (GBS) using a two‐sample Mendelian randomization (MR) approach.

**Methods:**

Publicly available summary data from genome‐wide association studies (GWAS) were utilized for comprehensive analysis. A genome‐wide and human leukocyte antigen association study conducted by Guillaume Butler‐Laporte et al. (*n* = 9724) examined 46 types of antibody‐mediated immune responses. GWAS summary statistics for GBS were obtained from the FinnGen consortium (*n* = 215,931) comprising European populations. The primary method for MR analysis was the inverse‐variance weighted (IVW) method. Various sensitivity analyses were conducted to assess the heterogeneity and pleiotropy of the findings.

**Results:**

The IVW method indicates a negative correlation between elevated levels of Epstein–Barr virus (EBV) viral capsid antigen (VCA) p18 antibody and the risk of GBS (OR = 0.79, 95% confidence interval [CI]: 0.65–1.85, *p* = 0.012). Elevated levels of *Toxoplasma gondii* surface antigen 1 (sag1) antibody also show a negative correlation with the risk of GBS (OR = 0.79, 95% CI: 0.67–0.92, *p* = 0.003). No evidence of heterogeneity or horizontal pleiotropy was found in the MR analysis.

**Conclusions:**

Elevated levels of EBV VCA p18 and *T. gondii* sag1 antibodies appear to be negatively correlated with the risk of GBS, suggesting that immune responses to these pathogens may play a protective role. However, the CI for the EBV VCA p18 association includes 1, indicating the need for caution in interpreting this result. Further research, including mechanistic studies and broader immune profiling, is needed to confirm these findings and explore the underlying pathways.

## Graphical abstract

Our objective is to investigate the potential causal relationship between antibody‐mediated immune responses to infectious and Guillain–Barré syndrome (GBS) by conducting a two‐sample Mendelian randomization (MR) study. In this MR study, we identified a negative correlation between Epstein–Barr virus VCA p18 antibody levels and GBS.

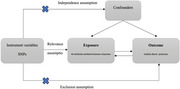

**Abbreviations**
EBV: Epstein–Barr virus
GBS: Guillain–Barré syndrome
GWAS: genome‐wide association studies
IVs: instrumental variables
IVW: inverse‐variance weighted
MR: Mendelian randomization
sag1
: surface antigen 1
SNPs: single‐nucleotide polymorphisms
VCA: viral capsid antigen

## 1 Introduction

Guillain–Barré syndrome (GBS) is the most common cause of acute flaccid paralysis worldwide and an immune‐mediated polyradiculoneuropathy, accounting for approximately 100,000 new cases annually (Sejvar et al. 2011). Most patients experience an acute onset of neurological symptoms following an infectious illness (Jacobs et al. 1998), leading to progressive limb weakness lasting up to 4 weeks before reaching a plateau. An autoantibody‐mediated immune process triggered by molecular mimicry between peripheral nerve components and microorganisms is well supported (Willison, Jacobs, and Van Doorn 2016). Despite standard immunotherapies, GBS remains serious, with around 5% mortality and up to 20% of patients unable to walk independently after one year (Shahrizaila, Lehmann, and Kuwabara 2021).

Infectious agents have been implicated in the pathogenesis of various noncommunicable diseases (O'Connor, Taylor, and Hughes 2006), including Alzheimer's dementia (Readhead et al. 2018), multiple sclerosis (Vanheusden et al. 2017), and GBS (Rodriguez et al. 2018). The immune response to infectious diseases is intrinsically linked to the human leukocyte antigen (HLA) system, which is encoded by the major histocompatibility complex gene complex (Blackwell, Jamieson, and Burgner 2009). Understanding immune responses to infection may help identify common pathways that, when disrupted, influence susceptibility to infections and immune responses (Butler‐Laporte et al. 2020).

Observational epidemiological studies often face biases, including confounding and reverse causation, limiting their ability to establish causal associations. Instrumental variables (IVs) approaches, such as leveraging germline genetic variants as proxies for modifiable exposures (Mendelian randomization [MR]), have been used to strengthen causal inferences in nonexperimental settings (Lawlor et al. 2008). This study aims to use MR to investigate the potential causal relationship between antibody‐mediated immune responses to infectious agents and the risk of GBS, offering novel perspectives for GBS intervention.

## 2 Materials and Methods

### 2.1 Study Design

**FIGURE 1:**
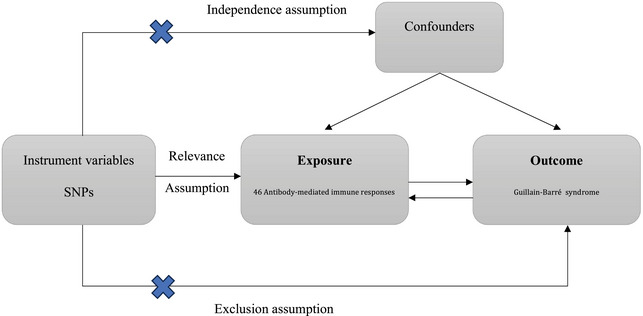
Assumption and design for the MR study. MR, Mendelian randomization; SNPs, single‐nucleotide polymorphisms.

Assumption 1 posits a substantial association between genetic variants and 46 types of antibody‐mediated immune responses. Assumption 2 entails that these genetic variants are unrelated to confounding factors influencing the exposure‐outcome association. Assumption 3 hypothesizes that the genetic variants influence GBS risk exclusively through their effect on antibody‐mediated immune responses. Figure 1 presents a brief description of this MR design.

### 2.2 Exposure Data

Forty‐six types of antibody‐mediated immune responses to infectious agents were investigated in a genome‐wide and HLA association study by Butler‐Laporte et al. (2020). They used data on 13 pathogens to define 46 phenotypes: 15 seropositivity case–control phenotypes and 31 quantitative antibody measurement phenotypes.

### 2.3 Outcome Data

Genome‐wide association studies (GWAS) summary statistics for GBS were obtained from the FinnGen consortium, focusing on the phenotype “Guillain–Barré Syndrome” (finn‐b‐G6_GUILBAR). The GWAS comprised 215,931 adult Finnish subjects (213 cases, 215,718 controls). As all data were publicly accessible, ethical endorsement or patient consent was unnecessary for the analysis.

### 2.4 Genetic Variants Selection Criteria

To investigate the causal relationship between antibody‐mediated immune responses and GBS, IVs were selected using a systematic approach. Initially, single‐nucleotide polymorphisms (SNPs) meeting the threshold for genome‐wide significance (*p* < 5 × 10^−5^) and demonstrating associations with the exposure were designated as potential IVs. Subsequently, a refinement process based on linkage disequilibrium (LD) was implemented, taking into account parameters such as *r* ^2^ and a window size of 10,000 kb (where *r* ^2^ < 0.001). This process ensured that selected IVs were independent. Furthermore, *F*‐statistics were calculated to assess the strength of the IVs, with a threshold set at *F*‐statistics > 10, a commonly recommended criterion for MR analysis. This rigorous selection procedure aimed to mitigate bias from weak instrument effects and establish robust causal inference between antibody‐mediated immune responses and GBS.

### 2.5 Statistical Analysis

The statistical analyses were performed using R (version 4.3.2) statistical software. MR analyses were conducted using the R package TwoSampleMR. The results of MR analyses were presented as odds ratios indicating the risk of the outcome per unit change in exposure, accompanied by respective 95% confidence intervals (CIs).

For the MR analysis, the *F*‐statistic was calculated to measure the strength of each IV (Burgess and Thompson 2011). IVs with *F*‐statistics > 10 were deemed robust instruments capable of mitigating bias typically associated with weak instruments. (Pierce, Ahsan, and Vanderweele 2011). The *F*‐statistic was calculated using the following formula: *F* = *R* ^2^ (*n* − 2)/(1 − *R* ^2^), where *n* is the GWAS sample size for the exposure association and *R* ^2^ is the proportion of variance explained by the genetic variants.

For the primary analysis, we employed the IVW method to estimate the impact of antibody‐mediated immune responses on GBS. This method provides concise estimates while addressing potential heterogeneity across individual variants. However, since the IVW method requires all genetic variants to be valid IVs for precise estimates, we complemented it with the MR‐Egger and weighted median approaches to evaluate its robustness (Bowden et al. 2016; Hartwig et al. 2016).

Heterogeneity was evaluated using Cochran's *Q* statistic and its associated *p* value (Verbanck et al. 2018). Following the assessment of heterogeneity using Cochran's *Q* statistic and associated *p* value, the results were visualized using funnel plots. Cochran's *Q* statistic was computed using the mr_heterogeneity function within the TwoSampleMR package. A *p* > 0.05 indicated no significant heterogeneity among the genetic IVs for antibody‐mediated immune responses. In addition, employing the leave‐one‐out method, any outliers among the SNPs were excluded, and the MR analysis was repeated to reinforce the robustness of the findings.

MR‐Egger intercept analysis was performed to investigate potential directional pleiotropy and determine whether the intercept of the MR‐Egger analysis was consistent with zero (Verbanck et al. 2018). The MR‐PRESSO test was employed to identify outlier variants exhibiting horizontal pleiotropy (Greco M et al. 2015). The MR‐Egger intercept and MR‐PRESSO tests were conducted using the mr_pleiotropy and mr_presso functions, respectively, within the TwoSampleMR package in R. A *p* > 0.05 indicated no evidence of pleiotropy among the genetic IVs for antibody‐mediated immune responses.

## 3 Results

### 3.1 MR Analysis of Antibody‐Mediated Immune Responses on GBS

**FIGURE 2:**
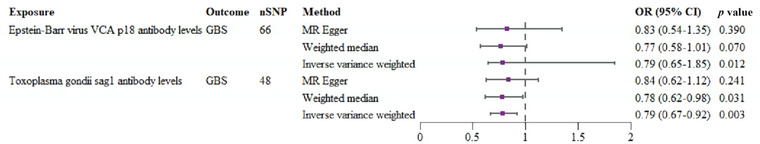
Forest plot for the causal effect of antibody‐mediated immune responses on the risk of GBS. CI, confidence interval; GBS, Guillain–Barré syndrome; MR, Mendelian randomization; nSNP, number of single‐nucleotide polymorphism; OR, odds ratio.

**FIGURE 3:**
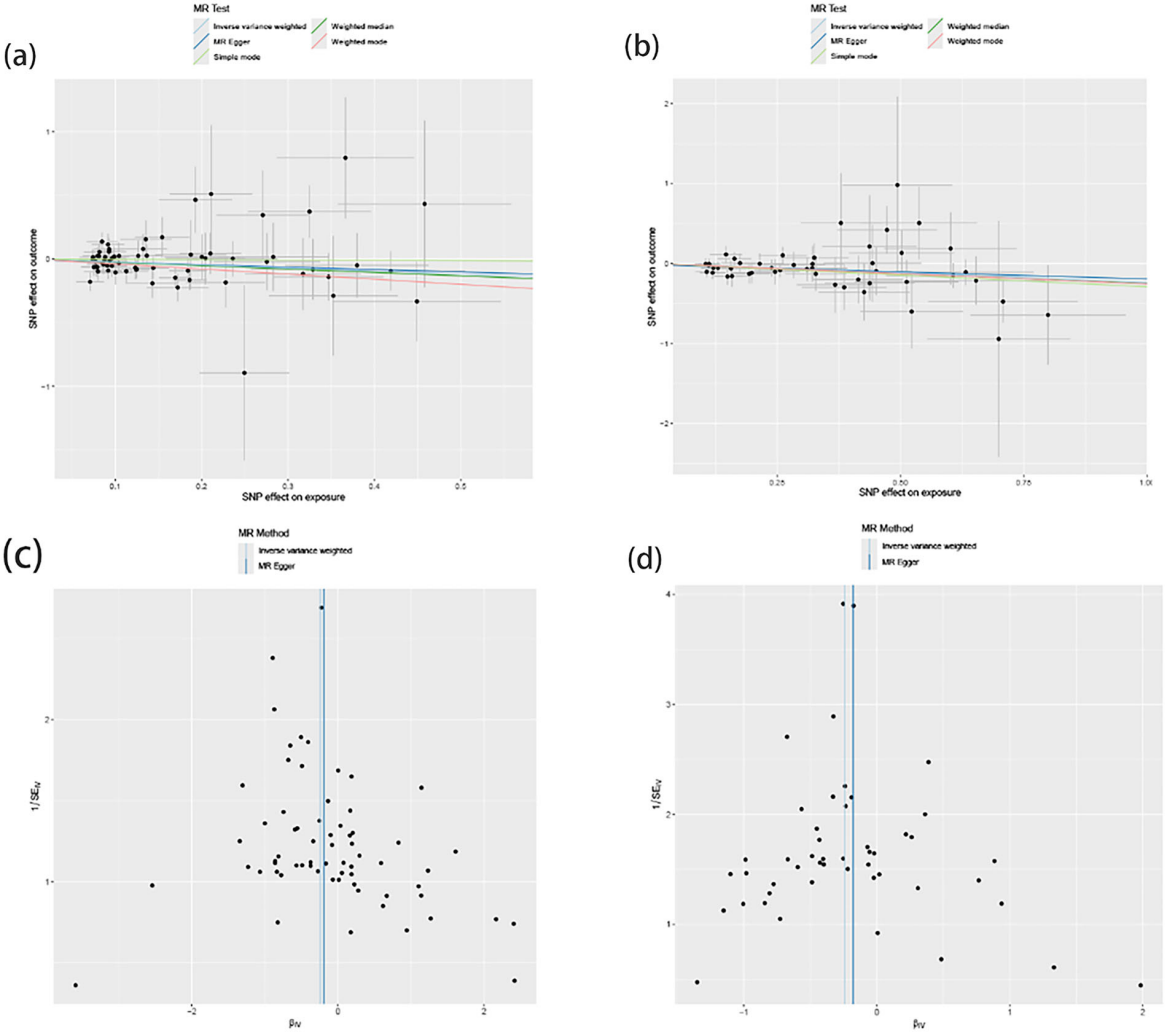
MR estimates from antibody‐mediated immune responses for GBS. (a) Scatter plots illustrate the significant associations between genetically predicted EBV VCA p18 antibody levels and GBS, with or without global weighting. (b) Scatter plots illustrate the significant associations between genetically predicted *Toxoplasma gondii* sag1 antibody levels and GBS, with or without global weighting. (c) Funnel plots illustrate the significant estimates from genetically predicted EBV VCA p18 antibody levels on GBS, with or without global weighting. (d) Funnel plots illustrate the significant estimates from genetically predicted *T. gondii* sag1 antibody levels on GBS, with or without global weighting. EBV, Epstein–Barr Virus; GBS, Guillain–Barré syndrome; MR, Mendelian randomization.

This study employed MR analysis to investigate the causal relationships between antibody‐mediated immune responses and GBS. The findings are shown in Figures 2 and 3. The IVW analysis revealed a statistically significant association between certain antibody‐mediated immune responses and GBS. Specifically, elevated levels of EBV VCA p18 and *Toxoplasma gondii* sag1 antibodies exhibited a negative correlation with GBS risk. For each standard deviation increase in genetically determined “EBV VCA p18 antibody levels,” the risk of GBS decreased by 21% (OR = 0.79, 95% CI: 0.65–1.85, *p* = 0.012; Figure 3a). Elevated *T. gondii* sag1 antibody levels also negatively correlated with GBS risk (OR = 0.79, 95% CI: 0.67–0.92, *p* = 0.003; Figure 3b). No statistically significant associations were found between 44 other antibody‐mediated immune responses and GBS.

### 3.2 Sensitivity analysis

To comprehensively investigate the correlation between EBV VCA p18 antibody levels and GBS, as well as *T. gondii* sag1 antibody levels and GBS, we conducted analyses to assess heterogeneity and pleiotropy (Figure 3; Table 1). Our findings indicate no statistically significant evidence of horizontal pleiotropy in the relationship between EBV VCA p18 antibody levels and GBS (Egger intercept = −0.007, *p* = 0.799). Similarly, this holds true for the relationship between *T. gondii* sag1 antibody levels and GBS (Egger intercept = −0.018, *p* = 0.625).

**TABLE 1:** Heterogeneity and horizontal pleiotropy of the associations between antibody‐mediated immune responses and the risk of GBS.

Exposure	Outcome	Heterogeneity				MR‐PRESSO	Pleiotropy test	
		Inverse‐variance weighted		MR‐Egger		*p* for global test	MR‐Egger	
		*Q*	*Q*_pval	*Q*	*Q*_pval		Egger_intercept	*p* value
EBV VCA p18 antibody levels	GBS	56.981	0.750	56.916	0.722	0.754	−0.007	0.799
*Toxoplasma gondii* sag1 antibody levels		27.501	0.990	27.259	0.987	0.990	−0.018	0.625

Abbreviations: EBV, Epstein–Barr virus; GBV, Guillain–Barré syndrome; MR, Mendelian randomization; *Q*, Cochran's *Q*; sag1, surface antigen 1.

No evidence of heterogeneity was found for EBV VCA p18 (Cochran *Q* = 56.981, *p* = 0.750; Figure 3c) or *T. gondii* sag1 (Cochran *Q* = 27.501, *p* = 0.990; Figure 3d) and GBS. MR‐PRESSO analysis further mitigated heterogeneity concerns (EBV VCA p18, *p* for global test = 0.754; *T. gondii* sag1, *p* for global test = 0.990). Funnel plots and line graphs supported the sensitivity analysis.

## 4 Discussion

Due to the presence of temporal causal relationships and confounding factors, traditional research methods often struggle to fully elucidate the complex relationship between antecedent infections and GBS. Exploring this relationship through the lens of host genetic variations has emerged as a crucial research avenue. In this two‐sample MR study, our results showed a statistically significant inverse association between elevated EBV VCA p18 antibody levels and GBS risk (OR = 0.79, 95% CI: 0.65–1.85, *p* = 0.012). Similarly, elevated levels of *T. gondii* sag1 antibodies also showed a negative correlation with GBS risk (OR = 0.79, 95% CI: 0.67–0.92, *p* = 0.003). These findings suggest that past immune responses to these pathogens may contribute to a reduced risk of developing GBS, possibly through immune‐modulatory mechanisms that warrant further exploration. However, caution is needed in interpreting the EBV‐related findings, as the CI includes 1. This suggests that the protective effect observed in our study may require additional validation in larger studies or more diverse populations. Sensitivity analyses, including MR‐Egger and MR‐PRESSO, did not reveal significant heterogeneity or pleiotropy, reinforcing the robustness of the results.

GBS is a multifactorial disorder, with contributions from genetic, environmental, and immune‐mediated factors. Genetic predisposition, such as specific HLA alleles, has been shown to influence susceptibility to GBS (Fu et al. 2024). Environmental factors, including viral infections such as EBV and *T. gondii*, can trigger immune responses that contribute to the onset of GBS (Karsten et al. 2022). In addition, vaccinations have occasionally been linked to GBS, although the incidence is rare, suggesting that immune‐mediated responses to environmental triggers may also be involved. The interplay between these factors and antibody‐mediated immune responses is crucial in determining the risk of GBS. Elevated antibody responses to pathogens like EBV may indicate a protective immune reaction, limiting viral replication and preventing the autoimmune mechanisms that trigger GBS (Islam et al. 2018). Understanding the complex interactions between these genetic, environmental, and immune factors will be critical for developing more effective prevention and treatment strategies.

GBS most commonly occurs following an infection, particularly viral infections, but it can also arise after immunization with certain vaccines or in association with the development of specific malignancies (Florian et al. 2021). Patients with GBS related to infections often produce antibodies against human peripheral nerve gangliosides through a process known as molecular mimicry (Ang et al. 2002). The cross‐reactive antigens of the EBV, recognized by macrophages and T cells, stimulate B cells to produce an anti‐ganglioside response. These antibodies penetrate the blood‐nerve barrier and activate the complement system. They bind to both gangliosides on peripheral nerves and antigens from microbes. Subsequently, activated endoneurial macrophages release cytokines and free radicals, invade the compact myelin and periaxonal space, and can obstruct nerve conduction or cause axonal degeneration. In addition, activated T cells release proinflammatory cytokines, fix complement, and damage Schwann cells, ultimately leading to the dissolution of myelin (Nyati and Nyati 2013).

Previous studies have established a potential link between EBV infections and autoimmune diseases such as multiple sclerosis, systemic lupus erythematosus, and GBS. The most widely accepted mechanism involves molecular mimicry, where immune responses to viral antigens resemble host neural tissue, leading to autoimmune attacks. Specifically, viral capsid antigens like EBV VCA p18 have been found to elicit strong immune responses, including IgG and IgA antibodies, which could potentially cross‐react with host proteins and trigger autoimmunity (Lim et al. 2015).

Our study, however, found an inverse relationship between elevated EBV VCA p18 antibody levels and GBS risk, which appears to contradict the idea that strong immune responses to EBV increase the risk of autoimmune diseases like GBS. Ferraro et al. (2016) provided evidence supporting a connection between EBV and GBS by showing the presence of EBV‐specific oligoclonal IgM and IgG bands in cerebrospinal fluid from patients with GBS. These findings suggest that EBV infection could play a role in the acute immune response during GBS onset. However, the specific antibodies and their role in disease progression remain complex and may vary depending on the individual's immune status and genetic background. Our study's observation of a potentially protective effect of VCA p18 antibodies may reflect a modulating or even immune‐resolving effect. One possible explanation for this protective effect is that individuals with higher levels of VCA p18 antibodies may have mounted a more effective immune response to EBV, preventing the immune dysregulation that typically triggers GBS. Another possibility is that elevated antibody levels reflect a long‐term, stabilized immune response to past EBV infection, which may reduce the likelihood of an autoimmune reaction to nerve gangliosides. These hypotheses align with studies suggesting that the timing and nature of the immune response to EBV play a critical role in determining whether the virus acts as a trigger for autoimmune diseases (Damania, Kenney, and Raab‐Traub 2022). Meanwhile, Budiningsih et al. (2023) suggest that elevated EBV‐specific antibody responses indicate a controlled immune response to the virus, preventing an autoimmune cascade. This immune control may limit viral load and reduce the chances of immune system malfunctions leading to GBS. Moreover, previous studies have indicated that higher antibody titers are associated with better control over EBV and a lower risk of autoimmune diseases such as systemic lupus erythematosus (Karsten et al. 2022). Higher antibody levels might, therefore, serve as protective markers, indicating a more balanced immune response. However, this finding requires further exploration.

*T. gondii* is an intracellular obligate parasitic protozoan that is globally distributed, has a wide host range, and causes zoonotic parasitosis. (Nardoni et al. 2011). *T. gondii* is an opportunistic infectious agent and may cause death in individuals with compromised or suppressed immune functions (Wang and Yin 2014). *T. gondii* infections cause substantial morbidity and mortality worldwide, with a wide spectrum of clinical manifestations in both immunocompromised and immunocompetent hosts (Layton et al. 2023). There are few publications of severe cases in immunocompetent patients due to scarce and very virulent strains. Cases of GBS following infection with *T. gondii* remain relatively rare, mostly represented by sporadic individual reports (Bossi et al. 1998; González et al. 2001; Satostegui et al. 2022).

The tachyzoite serves as the primary pathogenic form of *T. gondii*. In the acute phase of infection, tachyzoites trigger a robust host immune response that effectively clears a majority of the parasites. The surface of these tachyzoites is a primary target of the host's immune defenses. Specifically, *T. gondii* tachyzoites are adorned with glycosylphosphatidylinositol‐anchored antigens, predominantly belonging to the sag1 or sag2 families (Boothroyd et al. 1998). sag1 plays a pivotal role as a prominent surface antigen in this context. It possesses exceptional antigenicity and immunogenicity, eliciting robust humoral and cellular immune responses against *T. gondii* infection (Pagheh et al. 2020). During infection, the predominant antibody response is directed against sag1 (Velge‐Roussel et al. 1994). Consistent with our research findings, numerous studies have demonstrated that monoclonal, polyclonal, and monospecific antibodies targeting sag1 effectively inhibit the infection of human fibroblasts and murine enterocytes. (Kasper and Khan 1993) Meanwhile, a seminal study by Seng et al. (2004) suggests that sag1 may be instrumental in eliciting protective immunity and serves as a pivotal antigen that promotes Th1‐based responses during *T. gondii* infection in mice. The study by Zhou et al. (2021) demonstrates that as an immunogen, sag1 can induce the secretion of tumor necrosis factor‐alpha through the S100A6‐Vimentin/PKCθ‐NF‐κB signaling pathway.

This study demonstrates several notable strengths. First, it represents the inaugural large‐scale MR investigation employing random genotype allocation across two distinct cohorts. This approach significantly bolsters our ability to reliably ascertain potential causal links between antibody‐mediated immune responses to infectious disease agents and GBS, thereby providing novel therapeutic avenues. Secondly, the comprehensive utilization of diverse MR analysis techniques effectively mitigates the impact of confounding variables, enhancing the study's internal validity. Finally, rigorous primary sensitivity analyses were conducted to rigorously assess the assumptions of the MR model, further fortifying the robustness and reliability of our findings.

Our study is subject to several limitations that warrant consideration. First, our approach involved a threshold filtering of IVs at a significance level of *p* < 5 × 10^−6^, which some may argue is relatively lenient. Second, our sample was homogenous, comprising individuals of European ancestry exclusively, which restricts the generalizability of our findings to other ethnic or racial groups. Meanwhile, EBV seroprevalence and the immune response to EBV can vary significantly across different ethnic and geographical groups. For instance, studies have shown that people of different ancestries may have different levels of exposure to EBV, as well as differing genetic susceptibilities to autoimmune diseases triggered by viral infections (Damania, Kenney, and Raab‐Traub 2022). Future studies should aim to replicate these findings in diverse populations to better understand the role of EBV in GBS across different genetic and environmental contexts. Third, the most immediate limitation of our findings is the CI for the OR of EBV VCA p18 antibody levels, which includes 1 (OR = 0.79, 95% CI: 0.65–1.85). Although the *p* value is statistically significant (*p* = 0.012), the CI suggests a possible null association, suggesting that the association might be weaker or less certain than initially suggested. In addition, the variability in the CI could also reflect differences in the immune response among individuals, genetic variability, or environmental influences, which have been shown to contribute to GBS susceptibility (Fu et al. 2024; Karsten et al. 2022). Thus, future research with larger sample sizes, refined statistical methods, or different study designs may be necessary to confirm whether the association is truly causal or whether the observed effect might be a result of random variation or other confounding factors. Meanwhile, GBS is a multifactorial disease, and while antibodies against EBV and other pathogens may contribute to disease development, they are likely only part of a more complex autoimmune process. Addressing these gaps in the literature could pave the way for new preventive and therapeutic strategies for GBS.

## 5 Conclusions

This study suggests that elevated antibody levels against EBV VCA p18 and *T. gondii* sag1 are associated with a reduced risk of GBS. While the results for EBV VCA p18 require cautious interpretation due to the CI including 1, further research is needed to validate these associations in diverse populations and explore the underlying biological mechanisms. These insights may eventually inform strategies for GBS prevention and treatment.

## Author Contributions

**Xiangjia Qi**: software, data curation, conceptualization, methodology, investigation, validation, formal analysis, visualization, project administration, resources, writing–original draft. **Liqian Gao**: formal analysis, visualization, investigation, software, methodology, data curation. **Lifeng Qi**: writing–review and editing, project administration, funding acquisition, supervision, validation.

## Ethics Statement

There were no patients directly involved in the overall process of our study. Our study is based on publicly available data only. All human studies included in this analysis were conducted according to the Declaration of Helsinki.

## Consent

All authors agreed to the publication of this article.

## Conflicts of Interest

The authors declare no conflicts of interest.

### Peer Review

The peer review history for this article is available at https://publons.com/publon/10.1002/brb3.70298.

## Data Availability

All data generated or analyzed during this study are included in this published article. Codes generated or used during the study are available from the corresponding author by request.
